# Feasibility, safety, and preliminary outcomes of a school-based intervention combining paced-breathing HRV biofeedback and transcutaneous auricular vagus nerve stimulation in adolescents

**DOI:** 10.3389/fnins.2026.1924550

**Published:** 2026-08-10

**Authors:** Ainara Aranberri-Ruiz, Unai Liberal Graña

**Affiliations:** Department of Basic Psychological Processes and Their Development, University of the Basque Country (UPV/EHU), Donostia-San Sebastián, Spain

**Keywords:** adolescents, feasibility, heart rate variability biofeedback, paced breathing, school-based intervention, transcutaneous auricular vagus nerve stimulation

## Abstract

The vagus nerve is a major neurophysiological pathway linking the brain and body, and schools provide a practical setting for delivering stress-regulation programs. This uncontrolled pilot study assessed the feasibility, safety, acceptability, and preliminary outcomes of a school-based intervention combining paced-breathing heart rate variability (HRV) biofeedback with transcutaneous auricular vagus nerve stimulation (taVNS). Seventeen vocational education and training students aged 15–17 years enrolled in six weekly individual sessions of up to 30 min using emWave® biofeedback and Parasym™ taVNS. Feasibility was assessed through recruitment, six-session completion, acceptability, and adverse events. Exploratory outcomes included STAI-State anxiety, hair cortisol concentration (HCC), breathing self-regulation, and acute HRV indices. Recruitment was 50.0% (17/34), and 14 participants completed all sessions (82.4%). Among the 14 participants who completed all six sessions, 13 completed the written satisfaction item: 12 rated the program 5/5 and one rated it 4/5 (M = 4.92, SD = 0.28). One mild, transient stimulation-related adverse event occurred and resolved after pausing and resuming at a lower intensity. STAI-State scores decreased from pre- to post-intervention, whereas HCC increased. In nine participants with paired HRV data from Sessions 1 and/or 2, SDNN, RMSSD, lnHF, and lnLF were lower during combined paced breathing + taVNS than during spontaneous-breathing rest. Because the study was small, nonrandomized, uncontrolled, and the active components were always delivered together, these changes cannot be attributed to the intervention or to either component independently. The findings support cautious conclusions about deliverability and tolerability and provide information for a controlled trial, rather than evidence of efficacy.

## 1 Introduction

Adolescence is a period of heightened vulnerability to emotional and behavioral difficulties. The World Health Organization estimates that one in seven people aged 10–19 years lives with a mental disorder, with anxiety disorders affecting approximately 4.1% of 10–14-year-olds and 5.3% of 15–19-year-olds (World Health Organization, 2025; UNICEF Innocenti, 2025). Poor mental health also carries a substantial social and economic burden across Europe (OECD/European Union, 2018). These figures underscore the need for preventive interventions that strengthen emotion regulation, are well tolerated, and can be delivered feasibly in real-world settings.

Schools are strategically positioned to deliver prevention-oriented interventions (Van Loon et al., 2020). The present study focused on adolescents enrolled in basic vocational education and training, a second-chance pathway for students who have disengaged from mainstream schooling and may experience cumulative stressors, including academic disengagement, socioeconomic adversity, and language barriers (Cerdà-Navarro et al., 2024). Students in these settings may be less likely to access conventional services, making school-based implementation particularly relevant.

The vagus nerve provides a major body–brain pathway through which visceral signals contribute to autonomic and affective regulation (Berthoud and Neuhuber, 2000; Bonaz et al., 2018; Zhao et al., 2022). During adolescence, maturation of prefrontal–limbic systems coincides with changes in arousal and autonomic regulation (Casey et al., 2008; Grimm et al., 2024; Vink et al., 2020). Reduced vagally mediated HRV has been associated with vulnerability to stress-related affective difficulties (Thayer et al., 2012). Slow paced breathing at approximately 0.10 Hz, or six respiratory cycles per minute, can entrain cardiovascular oscillations near the resonance frequency of the baroreflex (Bernardi et al., 2001; Lehrer and Gevirtz, 2014). HRV biofeedback can serve as a pedagogical scaffold that helps participants find and maintain this rhythm. Previous school-based work by our group reported improvements in emotional self-regulation and attention after paced-breathing HRV biofeedback programs in primary education (Aranberri-Ruiz et al., 2022, 2024).

The breath-centered intervention was combined with transcutaneous auricular vagus nerve stimulation (taVNS), a noninvasive neuromodulation approach delivered through an auricular electrode (Aranberri-Ruiz, 2024; Ellrich, 2019). Studies in adults have reported autonomic changes during taVNS, although findings are heterogeneous (Clancy et al., 2014; Burger et al., 2020; Geng et al., 2022). Evidence in adolescents remains limited (Koenig and Vöckel, 2024; Zaehle and Krauel, 2021). Combining paced breathing and taVNS is theoretically motivated because both may engage overlapping autonomic and interoceptive pathways (Critchley and Harrison, 2013; Frangos et al., 2015; Tian et al., 2023, 2024). However, the feasibility of the combined protocol in a real-world adolescent school setting was unknown.

Accordingly, the primary aim of this pilot study was to describe feasibility across recruitment, completion of the six-session protocol, acceptability, and adverse events, consistent with recommendations for pilot and feasibility studies (Eldridge et al., 2016). The secondary aim was exploratory: to estimate pre–post changes in state anxiety, breathing self-regulation, and HCC, and to describe acute HRV differences between spontaneous-breathing rest and the combined paced-breathing + taVNS condition. Because the study was uncontrolled and nonrandomized and both active components were delivered concurrently, it was not designed to establish efficacy or component-specific effects.

## 2 Materials and methods

### 2.1 Study design and timeline

This pilot study used a nonrandomized, sequential-cohort design. Seventeen students were allocated to one of two consecutive cohorts according to the severity of presenting academic, affective, and behavioral difficulties, as assessed by school leadership. Students perceived to have greater support needs were prioritized for the Early cohort (*n* = 8), which received the intervention between November and December 2023. The Late cohort (*n* = 9) received the same protocol between January and February 2024. Allocation was therefore nonrandom, and between-cohort comparisons are subject to selection bias.

Outcome data were collected at three time points. Baseline (T0) was assessed 1 day before Session 1 and comprised hair sampling for cortisol quantification, the STAI-State scale, and an *ad hoc* item on habitual use of breathing for self-regulation. Immediate post-intervention assessment (T1) occurred 1 day after Session 6 and repeated these measures. Follow-up (T2) was conducted 7 weeks after T1 and included a third hair cortisol sample. Within each intervention session, HRV was recorded during a 3-min resting baseline with spontaneous breathing and no stimulation and during a single active HRV segment in which paced breathing at 0.10 Hz and taVNS were delivered concurrently. No separate paced-breathing-only segment was administered or recorded.

### 2.2 Setting, recruitment, and participants

The study was conducted at AZITAIN-IMFPB (Municipal Institute for Basic Occupational Qualifications) in Eibar, Spain. The intervention design and implementation were supported by a 2023 Kutxa Fundazioa grant within a collaborative agreement between DEBEGESA, the Economic Development Agency of the Debabarrena region, and the University of the Basque Country (UPV/EHU). The study received ethical approval from the Research Ethics Committee for Research Involving Human Subjects at UPV/EHU (CEISH-UPV/EHU; Ref. M10720247082).

AZITAIN-IMFPB provides a second-chance route for adolescents who have disengaged from mainstream secondary education because of factors such as language barriers, repeated academic failure, or socioeconomic disadvantage (Sarceda-Gorgoso and Barreira-Cerqueiras, 2021). Chronic absenteeism, low motivation, and limited linguistic proficiency are common challenges in comparable vocational settings (Amores and Ritacco, 2015; Keijzer et al., 2022). School leadership identified a need for practical strategies to support stress regulation and behavioral stability.

At the start of the 2023/2024 academic year, all second-year students enrolled at the center (*N* = 34) received detailed information about the program purpose, procedures, time commitment, potential benefits, foreseeable risks, and their right to withdraw at any time without penalty. Inclusion criteria were: enrollment as a second-year AZITAIN-IMFPB student; age 15–17 years; written informed consent from the participant and a legal guardian; ability to attend six weekly individual sessions and the planned assessments; no self-reported cardiovascular disease; and no self-reported medication considered likely to materially interfere with autonomic outcomes. Exclusion criteria were failure to meet an inclusion criterion, a medical contraindication to noninvasive taVNS or breath-focused HRV biofeedback, or inability to follow the planned schedule.

None of the enrolled participants reported a medical condition or medication that precluded participation. Consent procedures followed institutional ethical requirements for research involving minors and the Declaration of Helsinki (World Medical Association, 2013). Available baseline characteristics are summarized in Table 1. Demographic data were available for 16 of the 17 enrolled participants in the retained enrollment workbook; the missing record was not imputed.

**Table 1:** Available baseline and sample characteristics.

Characteristic	Early cohort	Late cohort	Overall/available sample
Enrolled participants, *n*	8	9	17
Age, years, *M* (*SD*), range	15.50 (0.76), 15–17 (*n* = 8)	15.63 (0.52), 15–16 (*n* = 8 of 9)	15.56 (0.63), 15–17 (*n* = 16 of 17)
Sex, female/male, *n*	4/4	4/4 (*n* = 8 of 9)	8/8 (*n* = 16 of 17)
Baseline STAI-State, *M (SD)*	36.88	42.78	40.00
Baseline HCC, pg./mg, *M* (*SD*)	17.12 (3.56)	27.12 (7.18)	22.02 (6.06)
Baseline HRV descriptive data	Not available by cohort	Not available by cohort	Exploratory paired analysis: *n* = 9
Medication expected to affect autonomic outcomes, *n*	0	0	0
Noncompleters with pre-existing chronic absenteeism, *n*	1	2	3
Repeated academic year among noncompleters, *n*	1	2	3

HCC, hair cortisol concentration; HRV, heart rate variability; STAI-State, State scale of the State–Trait Anxiety Inventory. Age and sex were available for 16 participants; one Late-cohort demographic record was absent from the retained workbook and was not imputed. Baseline STAI-State values are descriptive means from the submitted analysis; participant-level standard deviations were not available. Cohort-specific baseline HCC descriptive values were not reported in the submitted analysis. A dash indicates unavailable data.

Completion was defined as attendance at all six weekly sessions. The three noncompleters had pre-existing chronic absenteeism.

### 2.3 Measures

#### 2.3.1 Hair cortisol concentration

Hair cortisol concentration (HCC) was used as an exploratory biomarker of cumulative hypothalamic–pituitary–adrenal axis activity over preceding weeks (Karlén et al., 2011). HCC is influenced by multiple biological, behavioral, and contextual factors and was therefore not treated as a direct measure of intervention efficacy.

Trained staff collected approximately 64 strands of hair from the posterior vertex region, cutting as close to the scalp as possible. This site was selected in accordance with hair-testing guidance and its relatively consistent growth rate (Cooper et al., 2012; Wenning, 2000; Kintz, 2017; Thomson et al., 2010). Samples were placed in sealed, labeled plastic envelopes and stored at room temperature until analysis at the Clinical Chemistry and Pharmacology Laboratory at Linköping University, Sweden.

The proximal 2.0-cm segment was analyzed. Assuming average hair growth of approximately 1 cm per month, this segment represents roughly 2 months of cumulative exposure. Consequently, the post-intervention segment may include both the intervention period and part of the pre-intervention period, and the follow-up segment may overlap with the post-intervention period. Hair was cut, weighed, frozen in liquid nitrogen, homogenized, extracted overnight in methanol, lyophilized, reconstituted, and analyzed by competitive radioimmunoassay, following the laboratory procedure described by Morelius et al. (2004) and Karlén et al. (2011).

#### 2.3.2 Heart rate variability

HRV was recorded using the emWave Pro® system (HeartMath LLC, 2023), with a photoplethysmographic sensor attached to the earlobe. The Coherence Coach® module paced breathing at 0.10 Hz, corresponding to six respiratory cycles per minute. The proprietary HeartMath coherence display was used only as real-time biofeedback and was not analyzed as an outcome. Recordings comprised a 3-min resting baseline with spontaneous breathing and no stimulation, followed by a single 3–5-min active HRV segment in which paced breathing and taVNS were delivered concurrently. No separate paced-breathing-only segment was administered or recorded. The photoplethysmographic sensor was attached to the earlobe and the stimulation electrode to the left tragus; although the devices did not directly contact one another, movement- or stimulation-related photoplethysmographic artifact cannot be excluded.

Inter-beat interval data were exported and processed in Kubios HRV Premium (Kubios, 2023; Tarvainen et al., 2014). Time-domain indices were the standard deviation of normal-to-normal intervals (SDNN) and the root mean square of successive differences (RMSSD). Frequency-domain indices were natural-log-transformed low-frequency power (lnLF; 0.04–0.15 Hz) and high-frequency power (lnHF; 0.15–0.40 Hz), following conventional short-term HRV bands (Task Force of the European Society of Cardiology and the North American Society of Pacing and Electrophysiology, 1996). The exact artifact-correction and interpolation settings used in the original analysis were not prospectively logged, which limits reproducibility and is acknowledged as a study limitation.

Because later sessions contained substantial missing data, the exploratory HRV analysis was restricted to the first two sessions. For the nine participants with paired resting-baseline and combined-condition data from Sessions 1 and/or 2, values were averaged within condition across the available session data before paired comparisons. Respiration was paced during the combined condition but was not independently recorded. Baseline therefore involved spontaneous breathing, whereas the combined condition involved breathing at 0.10 Hz with concurrent taVNS. At this rate, respiratory-related oscillations shift toward the LF range; consequently, HF and LF cannot be interpreted equivalently across conditions, and the observed differences cannot be attributed separately to paced breathing or taVNS (Laborde et al., 2017).

#### 2.3.3 Self-report and contextual measures

State anxiety was assessed with the State scale of the State–Trait Anxiety Inventory (STAI-State; Spielberger et al., 1983). Only STAI-State was analyzed; trait anxiety was not an outcome of the present study.

Acceptability was assessed at the end of the intervention with one written item, “Are you satisfied with the intervention?,” rated on a 5-point Likert scale from 1 (not at all) to 5 (very much). A separate yes/no item asked whether participants usually used breathing to calm themselves and was administered before and after the intervention. These were *ad hoc* items rather than a validated usability scale. At program onset, participants also received a notebook in which they could record abrupt nonnormative life events, such as bereavement or serious illness, and other relevant experiences. Notebook entries were used only for contextual monitoring and were not analyzed as a qualitative outcome.

### 2.4 Devices and stimulation parameters

Individual sessions were conducted in a quiet, temperature-controlled room on school premises with two chairs, a table, and a computer connected to the biofeedback and stimulation equipment. The emWave® Pro desktop system, version 2021, was used to record inter-beat intervals and guide breathing at approximately six cycles per minute (HeartMath LLC, 2023).

taVNS was administered with a CE-marked Parasym™ device (Parasym Ltd., London, UK) through a soft-silicone electrode placed on the left tragus. The device was operated in constant mode at 25 Hz with a pulse width of 250 μs, consistent with the manufacturer’s instructions for the Parasym system. Stimulation was continuous during the 3–5-min active segment. Intensity was individually titrated in 0.8-mA increments until the participant reported a clearly perceptible but comfortable tingling sensation below the discomfort threshold. The protocol did not set a fixed study-specific maximum current; the upper limit was the participant’s discomfort threshold. Session-level intensities were not systematically logged.

### 2.5 Procedure

The intervention comprised six weekly individual sessions delivered during regular class hours over six consecutive weeks. Sessions lasted approximately 25–30 min and did not exceed 30 min, except for the final visit, which included closure procedures. Baseline assessment occurred **1** day before Session 1, immediate post-intervention assessment **1** day after Session 6, and HCC follow-up **7** weeks later.

Each session followed three phases. (1) Neutral acclimation (7–10 min): the researcher shared a brief neutral anecdote while a 3-min resting HRV recording was obtained with spontaneous breathing and no stimulation. (2) Breath-focused biofeedback with concurrent taVNS (3–5 min): participants followed paced nasal breathing at 0.10 Hz using the Coherence Coach® module while taVNS was delivered continuously. This was the only active HRV recording segment; no separate paced-breathing-only segment was administered or recorded. (3) Regulatory consolidation (5–10 min): after sensor removal, the researcher facilitated a calm conversation centered on notebook entries and encouraged the use of slow breathing during nervousness, agitation, or stress. Sessions 1–5 included informal discussion and notebook review; Session 6 included the written end-of-intervention item and a closing debrief.

### 2.6 Statistical analysis and sample size

Analyses were exploratory and intended to describe feasibility and obtain preliminary estimates rather than establish efficacy. Feasibility outcomes were summarized using counts, proportions, means, standard deviations, and ranges. Missing data were handled using complete cases for each outcome, without imputation. STAI-State was analyzed using the available 2 (Session: pre vs. post) × 2 (Cohort: Early vs. Late) × 2 (Sex: female vs. male) mixed analysis of variance (ANOVA). HCC was analyzed using a 2 (Session: pre vs. post) × 2 (Cohort) mixed ANOVA, together with an exploratory independent-samples t test for the baseline cohort difference. For each of the nine participants with paired HRV data from Sessions 1 and/or 2, values were averaged within condition across the available session data, and the resulting participant-level resting-baseline and combined paced-breathing + taVNS means were compared using paired-samples *t* tests. Effect sizes are reported as partial eta squared (ηp^2^) for ANOVAs and Cohen’s d for t tests, with 95% confidence intervals where these were available from the retained analysis. Exact *p* values are reported; however, given the small sample, missing data, nonrandom allocation, absence of a control condition, and multiple exploratory outcomes, they are not interpreted as confirmatory evidence.

The breathing self-regulation item was binary. Because the complete paired 2 × 2 contingency table was not retained, the previously reported McNemar statistic was not included in the revised analysis. Only the verifiable descriptive count of participants who changed from “No” before the intervention to “Yes” afterward is reported.

The sample size (*N* = 17) was determined by logistical and pragmatic constraints, including the school calendar, institutional access to the target population, and recruitment across two consecutive cohorts within a fixed time window. Such pragmatic sample-size determination is common in early feasibility studies, whose purpose is to assess implementation and obtain preliminary estimates rather than provide a definitive test of efficacy (Eldridge et al., 2016). A *post hoc* sensitivity analysis for a paired-samples comparison with *n* = 17, *α* = 0.05, two-tailed testing, and 80% power indicated a minimum detectable effect of Cohen’s d = 0.72 (Faul et al., 2007). Smaller effects may therefore have gone undetected, and all estimates should be regarded as provisional (Cumming, 2014; Lakens, 2022).

## 3 Results

### 3.1 Feasibility, acceptability, and safety

**Figure 1:**
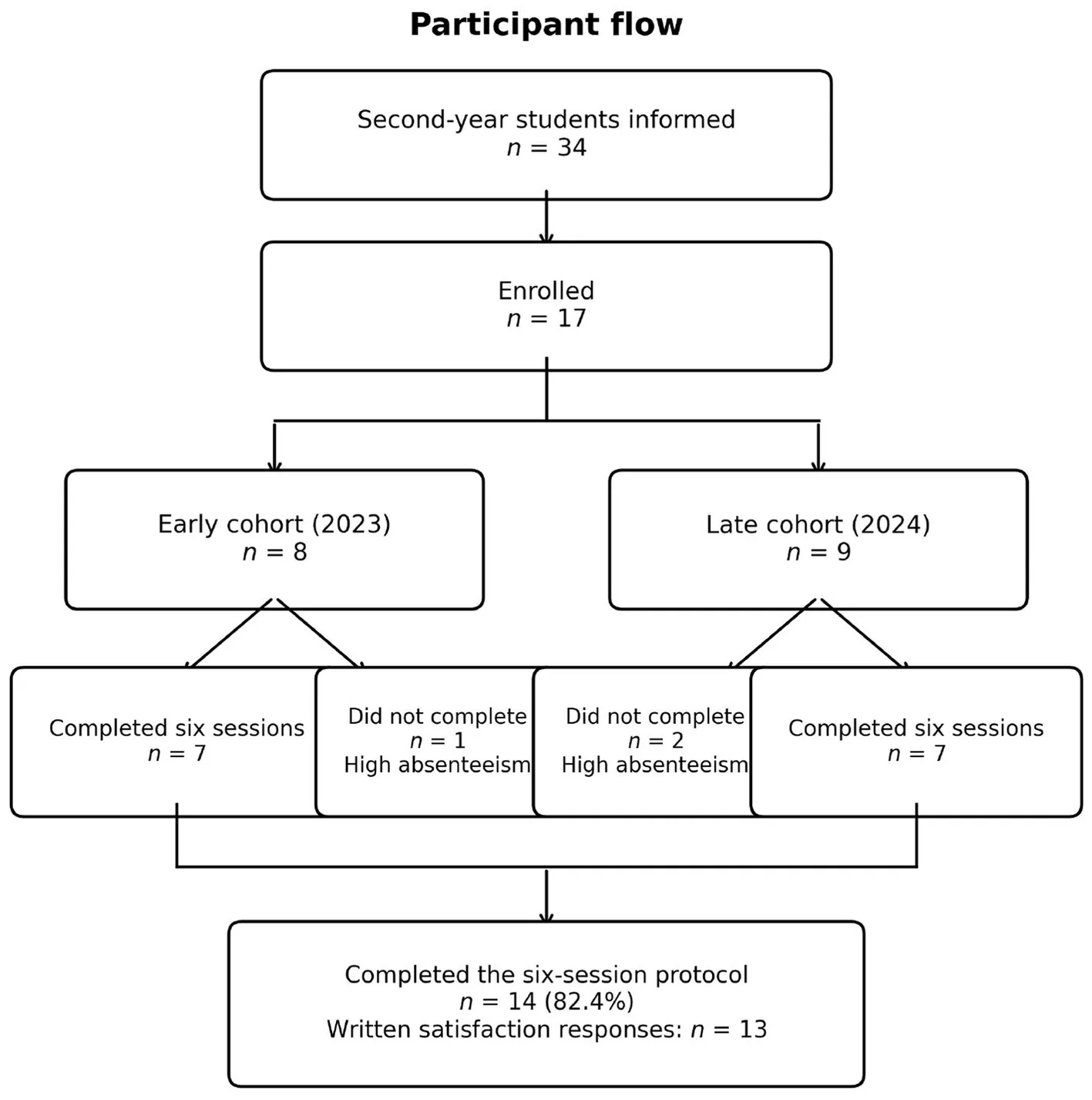
Participant flow.

The program was presented to all 34 s-year students, and 17 voluntarily enrolled, yielding a recruitment proportion of 50.0%. Fourteen participants completed all six sessions (82.4%): 7 of 8 in the Early cohort (87.5%) and 7 of 9 in the Late cohort (77.8%). The three noncompleters had a pre-existing pattern of high absenteeism and subsequently repeated the academic year; discontinuation was not attributed to discomfort or an intervention-related adverse event. Participant flow is shown in Figure 1.

Of the 14 participants who completed all six sessions, 13 completed the written satisfaction item. Twelve selected 5 and one selected 4, corresponding to *M* = 4.92, *SD* = 0.28, and a range of 4–5. No validated usability scale or systematic qualitative interview was administered. The contextual notebooks contained no reports of bereavement, serious illness, or comparable nonnormative events; entries referred only to routine changes in peer or romantic relationships typical of this developmental period.

One mild, transient adverse event occurred during taVNS. Immediately after the intensity had been increased through three 0.8-mA increments (2.4 mA), one participant reported a sudden electric-shock-like sensation. Stimulation was paused immediately. The participant reported having slept very little the previous night, required no medical assessment or additional intervention, and agreed to resume after a brief pause at one 0.8-mA increment (0.8 mA), without recurrence during the session. The event was classified as possibly related to taVNS because of its temporal association with the intensity increase. No other adverse events were recorded for taVNS or the emWave procedure.

These observations indicate that the combined protocol could be delivered and was generally tolerated in this setting. However, attendance-related attrition among students with chronic absenteeism limits broader claims of feasibility. Feasibility, acceptability, safety, and outcome-data availability are summarized in Table 2.

**Table 2:** Feasibility, acceptability, safety, and outcome-data availability.

Domain or outcome	Available data	Result or interpretation
Recruitment	17 of 34 eligible students	50.0% enrolled
Six-session completion	14 of 17 overall; 7 of 8 Early; 7 of 9 Late	82.4% overall; 87.5% Early; 77.8% Late
Written satisfaction item	13 of 14 completers	12 ratings of 5 and 1 rating of 4; *M* = 4.92, *SD* = 0.28, range = 4–5
Breathing self-regulation item	Complete paired denominator not retained	10 participants changed from No to Yes; descriptive only
STAI-State analysis	17 participants in submitted analysis	Overall *M* decreased from 40.00 to 35.71; exploratory mixed ANOVA
HCC analyses	Baseline cohort comparison: *n* = 16; pre–post model: *n* = 14	HCC increased at T1; interpretation is exploratory and noncausal
Exploratory HRV comparison	9 participants with paired data from Sessions 1 and/or 2	Resting values exceeded combined-condition values; respiratory confounding precludes a vagal interpretation
Adverse events	1 of 17 participants	Mild, transient event; stimulation paused and resumed at lower intensity; no medical follow-up

HCC, hair cortisol concentration; HRV, heart rate variability; STAI-State, State scale of the State–Trait Anxiety Inventory. The complete breathing-item contingency table and the exact follow-up HCC denominator were not retained and were not reconstructed.

### 3.2 Breathing self-regulation

Ten participants were recorded as changing from “No” before the intervention to “Yes” after the intervention when asked whether they usually used breathing to calm themselves. Because the complete paired 2 × 2 response table was not retained, the finding is reported descriptively and no McNemar test is presented.

### 3.3 State anxiety

**Figure 2:**
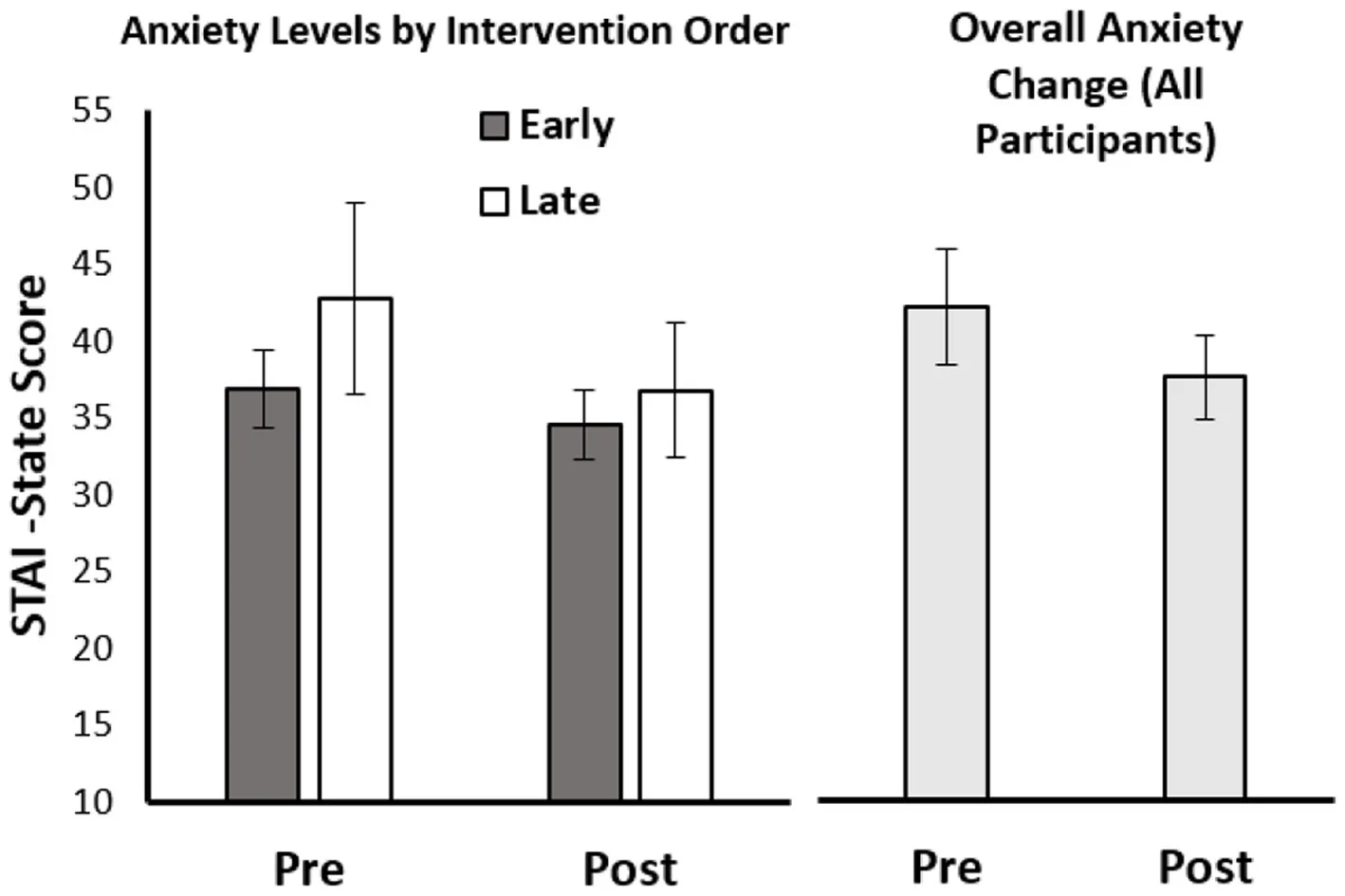
Descriptive STAI-State means before and after the intervention. **(A)** Shows cohort means; **(B)** Shows the overall sample mean. The bars display the standard error of the mean. The uncontrolled change should not be interpreted as evidence of efficacy.

Figure 2 presents descriptive STAI-State means before and after the intervention by cohort and for the sample as a whole. Mean scores were 36.88 at baseline and 34.50 post-intervention in the Early cohort, 42.78 and 36.78 in the Late cohort, and 40.00 and 35.71 overall.

The submitted mixed ANOVA yielded a main effect of Session, *F*(1, 13) = 6.79, *p* = 0.022, ηp^2^ = 0.34, 95% CI [0.039, 0.602]. Neither the Cohort main effect nor the Session × Cohort interaction reached the exploratory significance threshold (*ps* > 0.281). This uncontrolled pre–post change cannot be attributed specifically to the intervention and requires confirmation in a randomized controlled trial.

### 3.4 Hair cortisol concentration

One participant in the Late cohort did not provide a post-intervention sample and was excluded from the paired analysis. A mixed ANOVA with Session (pre vs. post) as a within-subject factor and Cohort (Early vs. Late) as a between-subject factor yielded a main effect of Session, *F*(1, 12) = 11.75, *p* = 0.005, ηp^2^ = 0.49, 95% CI [0.06, 0.703], reflecting higher HCC at T1. The Cohort main effect and interaction were not statistically significant.

An exploratory independent-samples comparison indicated a baseline difference between cohorts, *t*(14) = 2.14, *p* = 0.050, *d* = 1.10, 95% CI [0.00, 2.18], with higher baseline HCC in the Late cohort despite its classification as having comparatively lower presenting severity. Because allocation was nonrandom and groups differed at baseline, this comparison is not interpreted causally.

**Figure 3:**
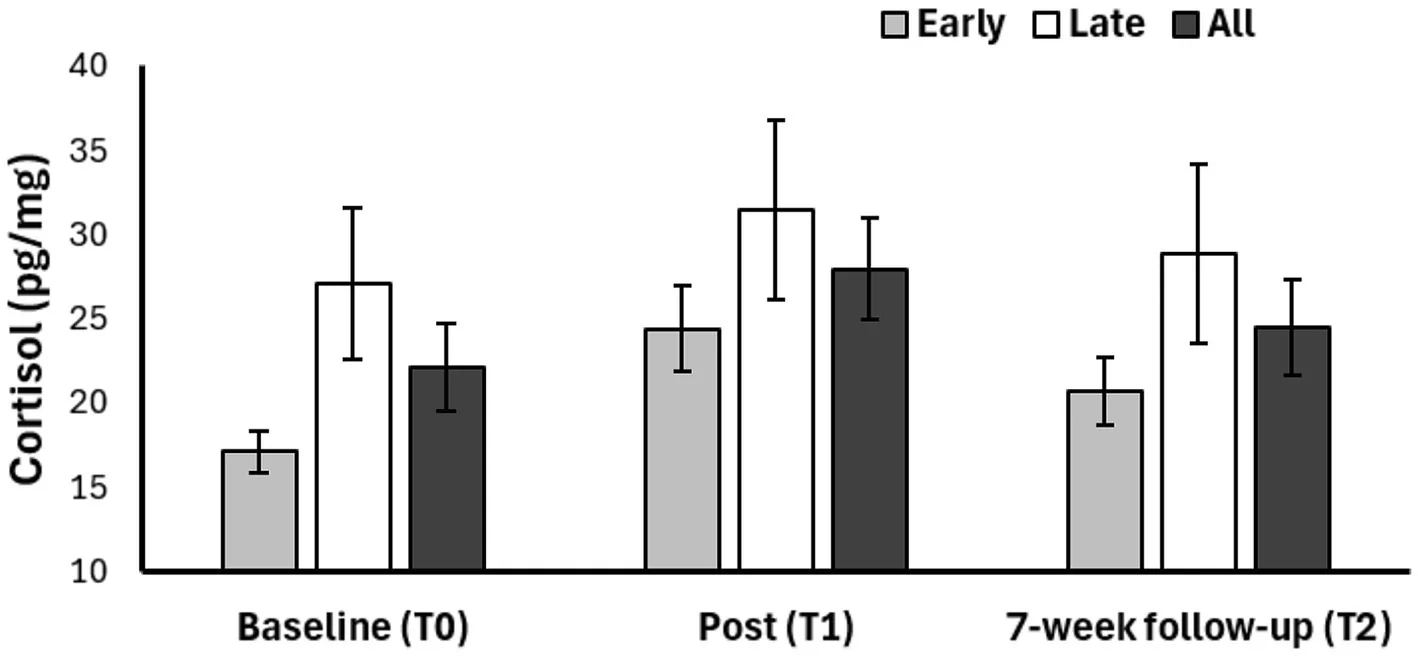
Hair cortisol concentration across the three assessment points. Bars show descriptive means and error bars show standard mean errors. T2 occurred 7 weeks after T1. The follow-up sample was reduced and no formal T2 comparison was retained. The 2-cm hair segments overlap in their retrospective exposure windows.

HCC was also collected at the seven-week follow-up. Descriptive mean values for the entire sample were 22.02 pg./mg at baseline (*SD* = 6.06), 25.51 pg./mg post-intervention (*SD* = 4.99), and 24.51 pg./mg at follow-up (*SD* = 5.31; Figure 3). No formal follow-up comparison was retained because of the reduced sample and absence of a control condition. Each 2-cm segment represents approximately 2 months of exposure, creating overlap between measurement periods. The observed pattern may reflect seasonal or school-related stress, baseline cohort differences, sampling limitations, or other uncontrolled factors.

### 3.5 Heart rate variability

Exploratory paired comparisons were conducted for the nine participants with paired resting-baseline and combined-condition data from Sessions 1 and/or 2. Values were averaged within condition across the available session data. Resting-baseline values were higher than values during combined paced breathing + taVNS for SDNN, RMSSD, lnHF, and lnLF (Table 3).

**Table 3:** Exploratory paired HRV comparisons: resting baseline versus combined paced breathing + taVNS.

Metric	Resting baseline *M* (*SD*)	PB + taVNS *M* (*SD*)	Difference *M* (*SD*)	*t*(8)	*p*	Cohen’s *d*
SDNN, ms	55.09 (33.21)	40.45 (30.91)	14.64 (15.50)	2.83	0.022	0.94 [0.13, 1.72]
RMSSD, ms	61.98 (36.07)	44.55 (36.84)	17.43 (21.34)	2.45	0.040	0.82 [0.04, 1.56]
lnHF, ln ms^2^	12.78 (2.24)	10.96 (1.64)	1.82 (2.14)	2.54	0.034	0.85 [0.06, 1.60]
lnLF, ln ms^2^	12.71 (2.63)	10.66 (2.13)	2.05 (2.42)	2.53	0.035	0.84 [0.06, 1.59]

PB, paced breathing; SDNN, standard deviation of normal-to-normal intervals; RMSSD, root mean square of successive differences; lnHF, natural-log-transformed high-frequency power; lnLF, natural-log-transformed low-frequency power. *n* = 9. Values were averaged within condition across the available data from Sessions 1 and/or 2. Difference and Cohen’s d are oriented as resting baseline minus PB + taVNS. These exploratory comparisons are confounded by the change from spontaneous to 0.10-Hz paced breathing and by concurrent taVNS. Confidence intervals are reported in brackets.

These comparisons should not be interpreted as evidence that the combined protocol reduced vagal activity. Baseline involved spontaneous breathing, whereas the active segment imposed a regular 0.10-Hz rhythm that changes the distribution of spectral power and the behavior of time-domain indices. In addition, paced breathing and taVNS were delivered together, so their separate or interactive contributions cannot be determined.

## 4 Discussion

This pilot study examined whether a combined paced-breathing HRV biofeedback and taVNS protocol could be delivered and tolerated in a real-world vocational school setting and described preliminary psychological, endocrine, behavioral, and autonomic estimates. The uncontrolled, nonrandomized design does not permit conclusions about efficacy or about the independent contribution of either intervention component.

### 4.1 Feasibility, acceptability, and safety

Recruitment was 50.0%, overall six-session completion was 82.4%, and written satisfaction among respondents was high. Completion was 87.5% in the Early cohort and 77.8% in the Late cohort. The three discontinuations occurred among students with chronic absenteeism and were not attributed to the intervention. This pattern suggests that the central implementation challenge was sustained attendance among students already at risk of disengagement. Feasibility was therefore not uniform across the full target population.

One mild, transient stimulation-related event required pausing and reducing intensity, with no medical follow-up. The event occurred immediately after an intensity increase and was classified as possibly related to taVNS. The available observations support cautious claims of deliverability and tolerability in this setting; they do not establish safety across broader adolescent populations or longer stimulation periods.

Acceptability was measured with a single *ad hoc* item rather than a validated usability scale. The high ratings among respondents are encouraging but should not be equated with comprehensive acceptability, particularly because one completer did not provide a rating and noncompleters were not represented.

### 4.2 Preliminary outcome estimates

The outcome findings are uncontrolled preliminary estimates and may reflect the intervention, researcher attention, repeated testing, school-calendar effects, seasonal influences, regression to the mean, or natural change over time.

#### 4.2.1 Breathing self-regulation

Ten participants were recorded as adopting breathing as a calming strategy from pre- to post-intervention. Because the complete paired response table was unavailable, this result remains descriptive and cannot be treated as a statistically established behavioral effect.

#### 4.2.2 State anxiety

STAI-State scores decreased from pre- to post-intervention. The direction of change is consistent with previous school-based breathing and biofeedback studies (Aranberri-Ruiz et al., 2022, 2024), but the absence of a control condition prevents attribution to the combined protocol. The result should be viewed as an estimate for planning rather than evidence of an anxiolytic effect.

#### 4.2.3 Hair cortisol concentration

HCC increased from baseline to post-intervention and remained descriptively above baseline at the seven-week follow-up. This finding is opposite to the hypothesized direction and should not be reframed as a beneficial physiological response. Notably, baseline HCC was higher in the Late cohort despite its classification as having comparatively lower presenting severity. This unexpected baseline difference further illustrates the selection bias introduced by nonrandom allocation and limits interpretation of the subsequent HCC changes. HCC and self-reported stress do not always correspond directly, particularly in small, heterogeneous samples (Gerber et al., 2012). Possible explanations include seasonal variation, school-related stressors, nonrandom baseline cohort differences, hair-sampling limitations, and temporal overlap of the 2-cm segments. Without a control condition and concurrent acute hypothalamic–pituitary–adrenal measures, the source and meaning of the increase cannot be determined.

#### 4.2.4 Heart rate variability

The exploratory HRV comparisons showed lower SDNN, RMSSD, lnHF, and lnLF during the combined condition than during rest. This pattern cannot be interpreted as a simple autonomic or vagal effect. At 0.10 Hz, paced breathing relocates respiratory sinus arrhythmia toward the LF range and imposes a more regular rhythm than spontaneous breathing. The comparison therefore conflates respiratory pattern with taVNS, and the short recording segments and incompletely documented preprocessing further limit interpretation. The study provides no evidence that taVNS independently altered HRV.

### 4.3 Limitations

Several limitations constrain interpretation. First, the study lacked paced-breathing-only, taVNS-only, sham, waitlist, and active-control conditions. Second, the sample was small and nonrandomly allocated, producing selection bias, baseline differences, and wide uncertainty. Third, three participants did not complete the protocol because of chronic absenteeism, highlighting implementation challenges among students with the greatest risk of disengagement. Fourth, acceptability was assessed with one *ad hoc* item rather than a validated scale.

Fifth, the HRV analysis was limited to nine participants and the first two sessions. Exact artifact-correction and interpolation settings were not prospectively logged; respiratory rate and depth were not independently measured; baseline and active conditions differed in breathing pattern; the photoplethysmographic sensor may have been affected by movement or auricular stimulation; and the 3–5-min segments were short for some frequency-domain analyses. Sixth, stimulation intensity was not logged at every session and the protocol used a discomfort threshold rather than a fixed numerical maximum.

Seventh, HCC segments overlapped in their retrospective time windows and may have been affected by seasonal, school, or sampling factors. Eighth, demographic records were available for 16 of 17 participants, and the complete contingency table for the breathing item and the exact follow-up HCC denominator were not retained. All inferential results should therefore be considered exploratory.

### 4.4 Implications for future research

Future research should use random allocation and a controlled design capable of separating the effects of paced-breathing HRV biofeedback and taVNS. A 2 × 2 factorial design crossing paced-breathing HRV biofeedback versus spontaneous breathing with active versus sham taVNS—or an equivalent dismantling design—would provide four conditions: spontaneous breathing with sham taVNS, paced-breathing HRV biofeedback with sham taVNS, spontaneous breathing with active taVNS, and paced-breathing HRV biofeedback with active taVNS. This design would allow estimation of the independent and interactive effects of both components (Collins et al., 2014). A waitlist or active-control condition could also be considered where feasible to address nonspecific pre–post changes.

Primary outcomes, stimulation settings, and HRV preprocessing procedures should be preregistered; respiratory rate and depth should be measured directly; HRV data and stimulation intensity should be logged consistently across all sessions; and acceptability should be assessed with a validated measure. HCC should be complemented by acute hypothalamic–pituitary–adrenal-axis measures and validated subjective measures of stress. Only a controlled design can determine whether taVNS provides incremental benefit beyond paced-breathing HRV biofeedback and whether any observed effects are attributable to either component or to their combination.

It should also be noted that taVNS requires specialist equipment, individual titration, and trained supervision, which constrains its scalability and generalizability relative to paced breathing alone; whether the added complexity and cost are justified by incremental benefit beyond the breathing component remains the central open question for future controlled work.

## 5 Conclusion

This uncontrolled, nonrandomized pilot study provides an initial characterization of the implementation, tolerability, and preliminary outcomes of combining paced-breathing HRV biofeedback with taVNS in a real-world vocational school setting. Half of the eligible students enrolled, 82.4% completed all six sessions, and satisfaction was high among those who completed the written item. One mild, transient adverse event, classified as possibly related to taVNS, occurred and resolved after stimulation was paused and resumed at a lower intensity. These observations indicate that the protocol could be delivered and was generally tolerated in this specific setting. However, attrition among students with pre-existing chronic absenteeism highlights an important implementation challenge, and the findings do not establish broader safety or comprehensive acceptability.

The exploratory findings showed different patterns across behavioral, psychological, endocrine, and autonomic domains: state anxiety decreased, 10 participants were recorded as adopting breathing as a calming strategy, HCC increased, and the analyzed HRV indices were lower during the combined condition than during spontaneous-breathing rest. These changes cannot be attributed to the intervention or to either component independently because the study lacked randomization and control conditions, paced breathing and taVNS were delivered concurrently, and the physiological analyses were affected by respiratory confounding, missing data, and incomplete procedural documentation. The present study therefore contributes primarily practical and methodological information for refining future school-based research. A sufficiently powered randomized controlled factorial or dismantling trial is required to determine the independent and interactive contributions of paced-breathing HRV biofeedback and taVNS and whether taVNS provides incremental benefit beyond paced breathing alone.

## Data Availability

The raw data supporting the conclusions of this article will be made available by the authors, without undue reservation.
